# inGeno – an integrated genome and ortholog viewer for improved genome to genome comparisons

**DOI:** 10.1186/1471-2105-7-461

**Published:** 2006-10-20

**Authors:** Chunguang Liang, Thomas Dandekar

**Affiliations:** 1 Department of Bioinformatics, Biocenter, University of Würzburg, Am Hubland, D-97074 Würzburg, Germany; 2 EMBL, Postbox 102209, D-69012 Heidelberg, Germany

## Abstract

**Background:**

Systematic genome comparisons are an important tool to reveal gene functions, pathogenic features, metabolic pathways and genome evolution in the era of post-genomics. Furthermore, such comparisons provide important clues for vaccines and drug development. Existing genome comparison software often lacks accurate information on orthologs, the function of similar genes identified and genome-wide reports and lists on specific functions. All these features and further analyses are provided here in the context of a modular software tool "inGeno" written in Java with Biojava subroutines.

**Results:**

InGeno provides a user-friendly interactive visualization platform for sequence comparisons (comprehensive reciprocal protein – protein comparisons) between complete genome sequences and all associated annotations and features. The comparison data can be acquired from several different sequence analysis programs in flexible formats. Automatic dot-plot analysis includes output reduction, filtering, ortholog testing and linear regression, followed by smart clustering (local collinear blocks; LCBs) to reveal similar genome regions. Further, the system provides genome alignment and visualization editor, collinear relationships and strain-specific islands. Specific annotations and functions are parsed, recognized, clustered, logically concatenated and visualized and summarized in reports.

**Conclusion:**

As shown in this study, inGeno can be applied to study and compare in particular prokaryotic genomes against each other (gram positive and negative as well as close and more distantly related species) and has been proven to be sensitive and accurate. This modular software is user-friendly and easily accommodates new routines to meet specific user-defined requirements.

## Background

Genomes are dynamic in nature and are known to undergo various types of changes in their evolution. Gene duplications result in paralogs, whereas gene deletions may induce loss of functionality. Recombination causes genome rearrangements, horizontal transfer introduces genetic materials into bacterial chromosomes, enabling the organism to recruit novel metabolic enzymes and consequently to survive in a different environment [1].

Early comparison methods to evaluate genome differences such as Needleman-Wunsch global alignment [2] and Smith-Waterman local alignment [3] were designed to identify sequence differences on a small scale. The methods use dynamic programming algorithms and have been proven to be sensitive to find an optimal alignment between sequences.

The increasing number of organisms whose genomes have been completely sequenced demands sensitive and precise methods for aligning long DNA sequences. Local alignments have been generally used to anchor global alignments. A variety of approaches such as MUMMER, WABA, AVID, Mauve and ACT [4-8] have been developed for genome visualization. Several other programs have been developed for specialized purposes, for instance, sim3 uses a greedy algorithm to investigate highly similar input sequences and works well even for long sequences [9]. The LAGAN series and MultiPipMaker (BLASTZ) are designed for dealing with genome rearrangements during the alignment process [10-12].

Here we describe a novel platform-independent genome comparison viewer, inGeno, which is user-friendly and modular. It recognizes and illustrates the functional relationships between orthologous genes and strain-specific genome islands. It accepts all major standard sequence input formats (Genbank, EMBL, Fasta, GenBankXML, SwissProt). It parses alignment reports (BLAST-type), performs a dotplot analysis, filters out the strain-specific genes of interest using a user-specified similarity threshold and plots a comparison map with an interactive interface (according to user choices, e.g. zooming in/out; genome representation style). The modules for information retrieval aid the user: Annotation keywords, logical combinations and concatenations of these, genome similarities and differences are identified from plain-text annotations and summarized and sorted by occurrences and functional categories (as color coded bars along the genome or as text reports).

## Implementation

### Strategy

Two genomes are aligned to each other and the resulting individual gene comparison outputs are parsed. This allows to establish a dotplot and correlation analysis, a linear regression on the orthologs and locus collinear block (LCB) locations in the genome. Furthermore, an interactive user interface for information retrieval enables to inspect detailed comparison results.

### Details

The program is completely implemented in Java and BioJava [13]. A Java runtime environment 1.5 is required to obtain platform-independency. A standard version of the program is available, based on an HSQL database (a relational database engine [14]) and a set of standalone tools written in Java. A further version integrates and requires a MySQL database engine previously installed on the system. This server-based version can be set up and handle a database suitable for long-term research purposes.

Sequence comparison data on individual genes are parsed using Biojava (re-implemented for different BLAST reports), by which the contig data, such as location, annotation and translated protein sequence information can be extracted efficiently from rich-format input files. A NCBI-BLAST interface and a Smith-Waterman-BLAST interface have both been implemented and are run for the user to prepare the data for the further analyses listed below.

InGeno interactively identifies and indicates the function and description of orthologous and strain-specific gene islands. Moreover, a keyword filter for annotation information aids the users (i) to investigate the genomes and (ii) writes different comparison reports (e.g., all pathogenicity factors in one genome in the region of interest).

- A dot-analysis algorithm is implemented to visualize the collinear ortholog relationships. Based on the coordinates, the program performs a linear regression and plots the regression line [examples are shown in Additional file 1]. Strain-specific genes and orthologous genes are determined according to a user-defined threshold. A combined threshold can be defined using multiple conditions, such as 75% alignment coverage and 30% sequence identity in parallel (our recommended combination).

- The log distances between the coordinates of each ortholog and the resulting regression line are then calculated and sorted. They are used in the next step for the heuristic determination of locus collinear blocks (LCB). Eventually the program provides an interactive graphical user interface (GUI), in which various operations are visualized. For example, each genome is represented as a horizontal, solid line. This can be shifted horizontally and vertically, zoomed in or out by sliders in the control panel. Genes of the same color linked by a line denote their orthology. These lines characterize the genome LCBs and indicate their distances (in colors). Their numbers and lengths can be re-defined by a slider in the control panel. By this, genome rearrangements can be viewed distinctly, including transpositions occurring in close or long distances.

- A text mining subroutine identifies functions of collinear genes parsing blast input and sequence input annotation: Tiny rectangles are plotted closely above the upper genome and below the lower genome. These are filled in different colors according to the parsed annotation files in order to differentiate the category of potential functions, e.g., blue denotes metabolism and enzymes, cyan denotes transcription/regulation factors and green denotes transporters/PTS systems (the color legend for this information appears by clicking the "function" button). This strategy improves operon recognition as well as the study of protein interactions. Another text-mining routine is implemented for the statistical analysis of strain-specific gene annotations. All the keywords are parsed, sorted by frequencies and subsequently filtered out to rapidly review the critical information related to different biological functions. Furthermore, there is the option to concatenate different keywords by logical operators (AND, NOT, OR) and in this way create more specific lists from the complete genome annotation. In addition, an editor is integrated in inGeno to correct annotation files and safe new annotation information.

- The program is implemented in Java for platform independency and in object-oriented programming (OOP) design. Different BioJava modules have been integrated. The whole structure is optimized to allow easy insertion of new modules and functional expansion if desired by the user.

## Results and Discussion

Required input data involve only the two annotation files and two sequence comparison reports (Blast or Smith and Waterman search results, see above), the inGeno software then compares the two sequence comparison reports to each other (reciprocal comprehensive comparisons of predicted protein versus predicted protein from both genomes). The user can select the alignment coverage (upper slider in bottom control panel; [see Additional file 1]) and the percentage of identity (lower slider) that similar proteins from the two genomes compared have to share to be shown as orthologs by the genome viewer. Only the best pair (by E-value) meeting these thresholds is shown by inGeno, a back-tracking procedure eliminates all paralogs meeting the threshold but having a lesser e-value.

Using this genome viewer, we have investigated a variety of genome sequences, such as comparisons between species within a genus (*Listeria *spp. or *Escherichia *spp.). The resulting alignment map is plotted and provides clues for new studies. Comparisons between organisms of larger phylogenetic distance (e.g., between different genus, *Bacillus *spp. and *Listeria *spp.) produces in general an alignment lacking conserved collinear relationships. However, interesting strain-specific islands and their specific functionalities, such as new recruited metabolic reactions or regulatory proteins and transcription factors are still easily detected by using our software.

The two annotation files supply the genome viewer with annotation information. To correct and prune wrong annotation, these files can be re-edited by the user within the program checking for pseudo-genes or bad annotations. The visualization of the genome including its annotation allows the user to go over the whole genome, correct important annotation mistakes, save all corrections made and then run inGeno again to see the result of the two corrected genome comparison. Furthermore, a GenomeToProteome routine [see Additional file 1] compiles from a DNA sequence which at least contains the start and end points of the reading frames all proteins in multi-fasta format and prepares them for systematic genome comparison with BLAST or Smith-Waterman-search program. The output files created after the search can again be directly parsed by inGeno [see Additional file 1] and so a comparatively raw DNA file can also be visualized with its annotation by inGeno, provided the BLAST runs are done by the user. However, inGeno focuses on displaying reading frames and features of the genomes (including even RNA genes or DNA features [e.g. IS element] if they are part of the annotation report or edited into it) but there is no direct visualization of DNA features, for this task other genome viewers, e.g. the Artemis Comparison Tool [8], should be used.

We stress that pseudo-genes have to be marked in the annotation as such if they are to be accurately visualized. However, the back-tracking procedure from inGeno automatically ensures that pseudo-genes that are less similar to the other genome than the best available intact gene copy are not shown as wrong orthologs.

InGeno allows a precise visualization of the alignment of the proteins and further annotated features between the two genomes. In related genomes collinear relationships are highlighted [see Additional file 2]. All routines are sensitive and based on the information extracted from orthologous and strain-specific genes.

The user has a number of options to visualize and identify similarities and differences between both genomes [details and tutorial examples are given in Additional file 1]. Orthologous proteins between both genomes are shown according to user specified thresholds regarding the percent identity and the length of similarity in both sequence alignment comparison files (genome 1 to genome 2 and genome 2 to genome 1) required to be operationally declared as an orthologue. A test routine ensures that only the best orthologous pair found to pass these thresholds is shown. Orthologous proteins in both genomes are indicated as ellipsoids in the same randomly chosen color. Strain specific genes stand out and are marked in red. After establishing the best regression line to compare both genomes, locus collinear blocks (similar nucleotide distance to the best regression line and consecutive to each other) identify gene regions which cluster together but are rearranged in the two genomes. Rainbow coloring of linkage lines between the two genomes indicates close synteny regions (red) up to major rearrangements between both genomes (blue). The coloring is either according to the distance to the regression line (*regression mode*; for close related genomes) or according to the absolute gene to gene distance for the two genomes are compared (*absolute mode*; for more distantly related genomes). Sliders position the genomes horizontally and vertically in the display, radio buttons switch colors, the number of linkage lines displayed and set user-specified thresholds. A "legend" option explains the different colors used in the display.

InGeno allows the user to search for string matches, e.g. keywords such as "kinase" from the annotation file. The user can select a region of interest from a genome (including the whole genome) and inGeno sorts the annotation keywords within the selection area according to their frequency. In this way the user gets an overview for key functions encoded in this area of the genome (e.g. indication for a cluster of pathogenicity factors). As an important help in detailed comparisons of the annotation files several keywords can be searched for at the same time and also be concatenated by logical operators (AND, NOT, OR). In this way also very complex queries and reports for strain specific features or for common features can be generated (e.g. a pathogenicity factor but not a kinase).

New information compiled for the user from the genome viewer by inGeno thus includes gene visualization, sorting and grouping as well as including importance of different keywords, string-matching and logical concatenation of terms used in the annotation text-mining routine, a graphical display of genome similarities, orthologous regions and strain-specific differences including strain specific islands and the creation of function specific annotation reports and lists.

A number of genome analysis and visualization tools are currently available and several of the specific advantages and limitations are discussed below.

The latest version of MUMmer [15] easily handles comparisons of large eukaryotic genomes at varying evolutionary distances. Two new graphical viewing tools provide alternative ways to analyze genome alignments. The new system is the first version of MUMmer to be released as open-source software [see Additional file 3].

MuGeN [16] explores multiple genomes and computer analysis results. It is capable of retrieving annotated sequences in several formats, stored in local files, or available in databases over the network. From these, it then generates an interactive display, or an image file, in most common formats suitable for printing, further editing or integrating in Web pages. Genome maps may be mixed with computer analysis results loaded from XML files.

Mauve [7] focuses on multiple alignments of a conserved genomic sequence with rearrangements. It has been applied to align nine enterobacterial genomes and to determine global rearrangement structure and evolution in three mammalian genomes.

Furthermore, the Artemis Comparison Tool (ACT, [8]) allows for an interactive visualization of comparisons between complete genome sequences and associated annotations. The comparison data can be generated with several different programs (e.g. BLASTN, TBLASTX or Mummer) or orthologue tables generated by reciprocal FASTA comparison between protein sets. ACT uses Artemis components to display the sequences and so inherits powerful searching and analysis tools. It is written in Java and platform independent.

Finally, in parallel to this work, recently a Java-based genome viewer appeared ("Combo", [17]) as an integrated part of the Argo Genome Browser which also provides single-genome browsing and editing capabilities and a dot-plot and genome annotation viewer.

All these tools have their pros and cons and reveal partly different information from the genome. Some of the differences and advantages of inGeno as a genome viewer are analyzed and summarized below.

InGeno shows several advantages which some of the existing software also has, e.g. open source, Java code [see Additional file 3] and platform independence (ACT and Combo) as well as interactive visualization of genome data (all above mentioned genome viewers). InGeno is not intended for simultaneous comparison of multiple genomes (in contrast to MuGen [16] and Mauve [7]). However, specific advantages are its modular construction using Biojava modules (easy further development in the community is possible) and its rich user interface [see tutorial and instructions in Additional file 1]. Only limited input is required: Two original sequence annotation files and two ordinary BLAST reports on the two complete genomes compared are sufficient.

To get a solid genome alignment the program starts with a dotplot (as Combo does) but then inGeno identifies locus collinear blocks (LCB) and includes as an inGeno-specific advantage a back-tracking procedure to clean up the alignment and show only true positives as orthologous proteins. Furthermore, inGeno allows for user specified thresholds [percent identity, length of the alignment, see Additional file 1] in the visualization of potential orthologs from both genomes (again including the back-tracking so that only best hits from both genomes are shown).

In addition, inGeno has an improved visualization compared to the available tools by user-specified coloring [see Additional files 1 and 2] such as linkage lines. We prevent cluttering of the view (threshold options, "hide" option) and allow a range of visualization options as well as detection of genome rearrangements (synteny up to major rearrangement) by the sensitive LCB method.

Furthermore, the protein annotation in the genomes is not only visualized for both genomes compared (as in Combo, MuGen) but actively processed so that reports for complex regular expression (e.g. ("kinases that are not pathogen-associated" OR "phosphatases")) can be compiled and visualized for both genomes. This is important to interpret for example the highlighted strain-specific islands which is not available in this form in the above other viewers. We thus think that the optimized, user-friendly view provided by inGeno is at least a good alternative for a two-genome comparison, notwithstanding the qualities of the alternative above-mentioned genome viewers.

In summary, our software allows rapid genome comparisons and gene visualization, gene sorting and grouping. Furthermore, the user is alerted on the importance of different keywords and string-matching from the text-mining routine, a graphical display of genome similarities, orthologous regions and strain-specific differences including highlighted strain specific islands as well as the creation of function specific annotation reports and annotation listings for complex queries

### Application examples

**Figure 1:**
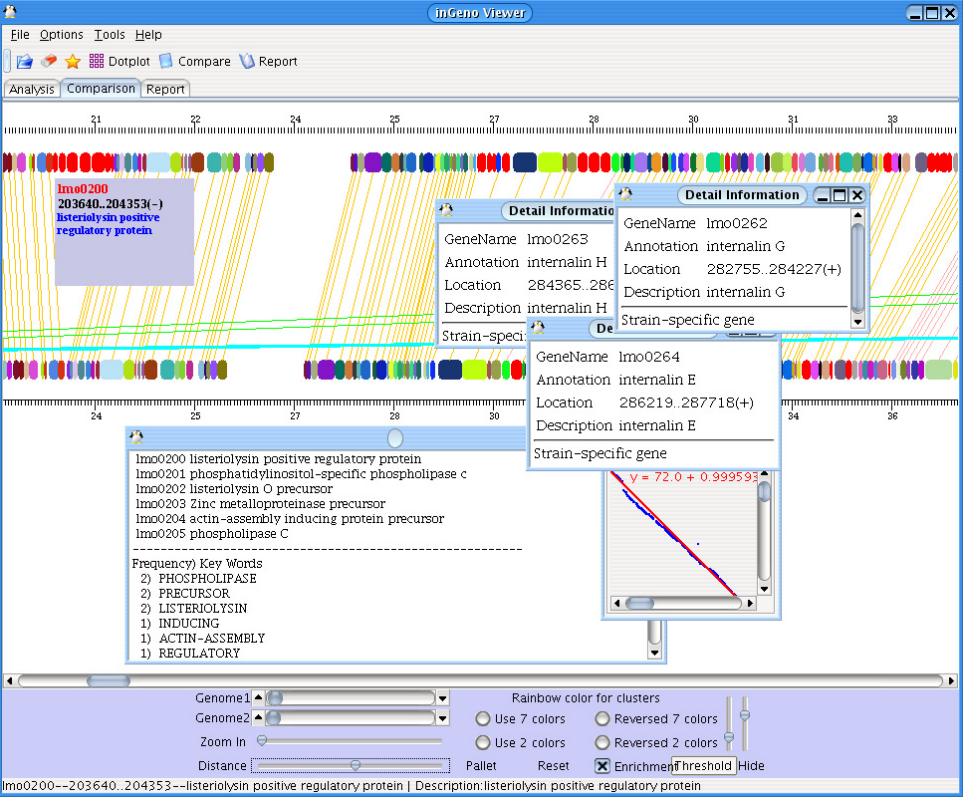
***Interactive graphical user interface for genome alignment***. Genomes of *Listeria monocytogenes *(lower genome in the figure) and *Listeria innocua *(upper genome) are compared using inGeno. Orthologous genes in both genomes are colored with the same color. Linkage lines connect locus collinear blocks and indicate the degree of rearrangement between the genomes. The threshold can be adjusted by a slider in the lower-right corner of the control panel. Red blocks in each genome distinguish genes which are potential strain-specific and determined by a user-given threshold. In this comparison several strain-specific genome islands are detected, e.g., in the figure a red island beginning with *lmo0200 *is being investigated. It is part of the Lipi1 pathogenicity island. Clusters of green lines indicate genome rearrangement events, these can be caused e.g. by transposons. A large number of transposase genes are found and visualized in the *L. monocytogenes *genome.

Figure 1 demonstrates several of these features in a typical comparison map, comparing *Listeria *genomes between *L. innocua *strain CLIP 11262 (Genbank accession: NC_003212) *and L. monocytogenes *stain EGD-e, (Genbank accession: NC_003210) [18]. The alignment of orthologs predicts a considerable conservation of proteins with similar function. Potential strain-specific genes are highlighted and colored in red (Fig. 1), they tend to cluster here as islands. They may have been introduced either by foreign genome segment adoptions, e.g. phage insertions, or by large genome deletions in the other organism. In addition, the program provides two options to view the associated annotations, simply by moving the mouse above the gene block or by double-clicking. The latter will open a window and report information on the gene in detail. The summary of the strain-specific gene reports includes here a couple of surface-associated and small secreted proteins which belong to the internalin family, i.e., Internalin B (*lmo0434*), Internalin C (*lmo1786*), Internalin G (*lmo0262*), Internalin H (*lmo0263*), Internalin E (*lmo0264*), and several other uncharacterized proteins containing LPXTG motifs. The investigation reveals a variety of metabolism-related enzymes absent in *L. innocua*, e.g., the genes involved in bile acid degradation (*lmo0446*, *lmo0754*, *lmo2067*), which are potentially the reason that *L. monocytogenes *has the capability of surviving in the gut. The genes which are part of the LIPI-1 [21,22] virulence cluster indispensable for the intracellular life style of *L. monocytogenes *such as PrfA (*lmo0200*), PlcA (*lmo0201*), Hly (*lmo0202*), Mpl (*lmo0203*) till phopholipase C (lmo 0205) are readily identified in the map. In accordance with earlier studies [18-21], these characterize *L. monocytogenes *as a food-borne pathogen and enable it to survive in multiple extreme conditions in vivo and in vitro [22].

**Figure 2:**
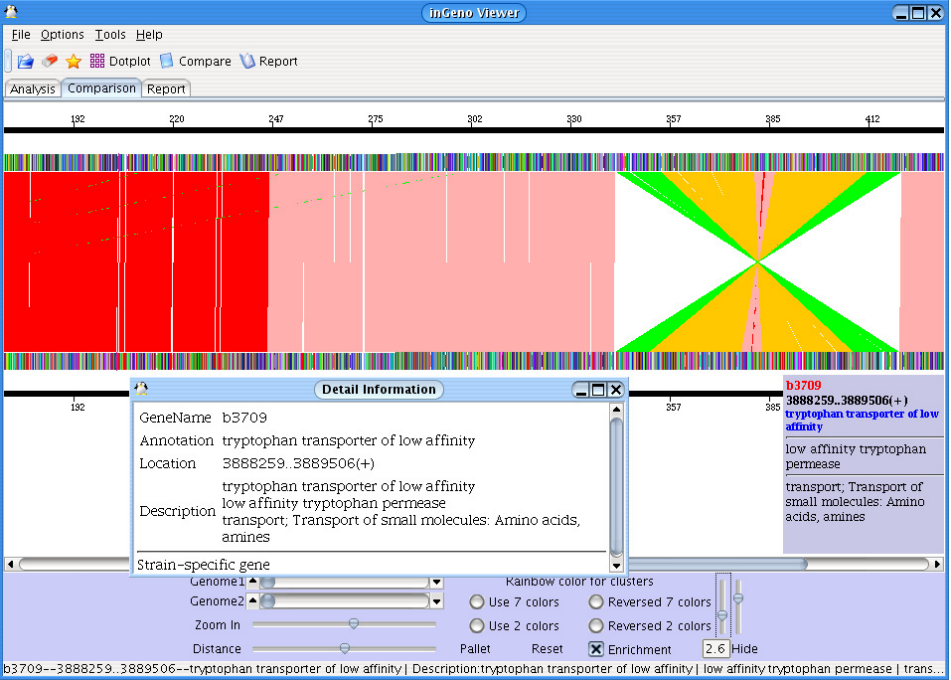
***Genome comparison between closely related strains***. The comparison between two closely related *E. coli *K-12 strains (W3110 and MG1655) indicates these are only slightly different, except for a highlighted large inversion. The upper genome is W3110 [24], whereas the lower genome is MG1655 [23-25]. Using inGeno, a couple of strain-specific genes are readily seen, such as TnaB (annotated as low affinity tryptophan permease; *b3709 *in figure) and other genes (DcuC: *b0621*, GatA: *b2094*, RcsC: *b2218*), which lead to different metabolic capabilities, e.g., the utilization of tryptophan as carbon source may be impaired in W3110.

Fig. 2 illustrates inGeno performance in a genome comparison between two closely related strains, i.e., *E. coli *K-12 strains MG1655 (Genbank accession: U00096) and W3110 (Genbank accession: AP009048) which immediately indicates that these two are extremely similar. Most genes are highly conserved, but there is a large inversion for W3110 in the region shown in Figure 2 close to the 3' prime terminus of replication. In accordance with recent studies [23], inGeno shows a number of IS element copies. InGeno further detects a couple of strain-specific genes, such as Dcuc (anaerobic C4-dicarboxylate transport: *b0621*), GatA (galactitol-specific enzyme IIA component of PTS: *b2094*), Rcsc (hybrid sensory kinase in two-component regulatory system with RcsB and YojN: *b2218*) and TnaB (low affinity tryptophan permease: *b3709*). They are present in MG1655 but pseudogenes in W3110 [24]. Each of these displayed variations may influence cell metabolism, for instance carbon utilization of tryptophan, galactitol or succinate.

**Figure 3:**
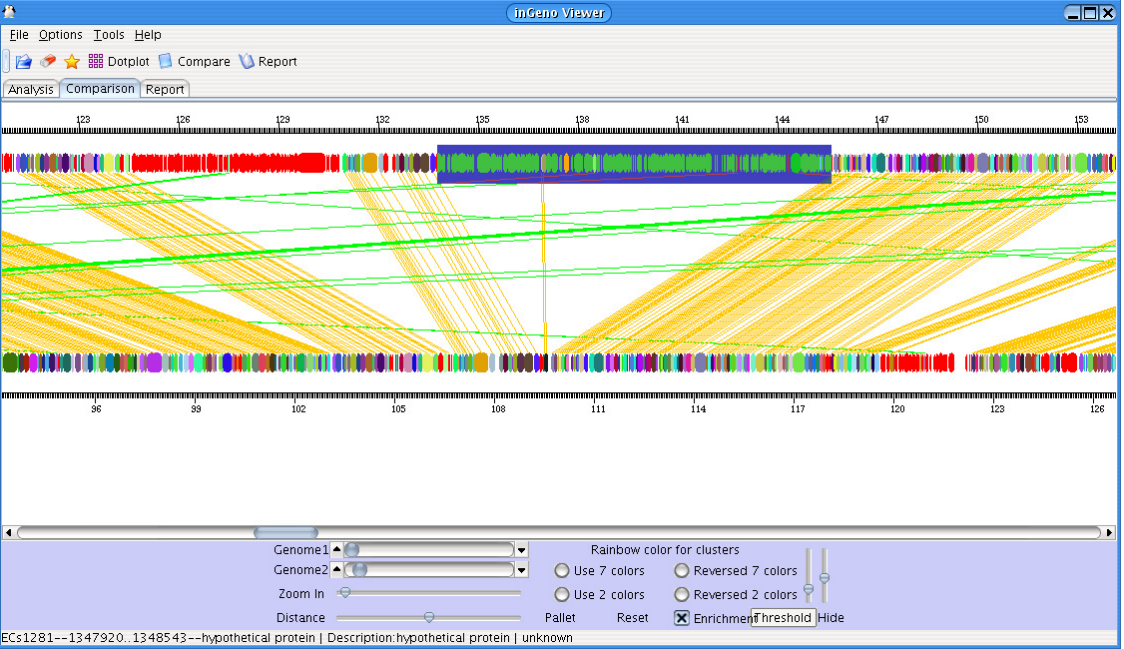
***Strain-specific island – investigation of pathogenicity and metabolism***. The top genome is *E. coli *O157 [25], the lower is *E. coli *K-12 strain MG1655 [23-25]. The selected region (ECs1272-1296 and ECs1299-1409) is one of the strain-specific islands that are potentially related to bacterial pathogenicity. ECs1282 and ECs1283 are identified by inGeno as hemagglutinin/hemolysin-like protein and hemolysin activator-related protein, respectively. An operon-like structure follows these two genes. InGeno reports these encode for a holo acyl-carrier protein, an oxoacyl-(acyl-carrier protein) reductase, a hydroxydecanoyl-(acyl-carrier protein) dehydratase, an acyl-carrier protein, an aminomethyl transferase and an oxoacyl-(acyl-carrier protein)-synthase. These enzymes and proteins add to the fatty acid metabolism, additional lipids or lipoproteins may be produced by O157 in contrast to MG1655. Moreover, a series of continuous genes encoding urease components are shown for 0157 (ECs1321-1327: UreA-G). The detailed information on these proteins is summarized in Table 1.

Analysis of pathogenicity features is demonstrated in Figure 3. These are inGeno results comparing *E. coli *strain O157:H7 (Genbank accession: BA000007) and *E. coli *strain MG1655. The *E. coli *strain O157:H7 is a major pathogen that causes diarrhea, hemorrhagic colitis and hemolytic uremic syndrome [25]. *E. coli *K12 strain MG1655 is chosen as an apathogenic control strain. InGeno reveals the strain-specific pathogenicity islands present in O157:H7, e.g. an island (location: 1337361–1456555), is predicted to be involved in bacterial pathogenicity and lipoprotein metabolism. InGeno reports that it encodes a hemagglutinin/hemolysin-like protein (*ECs1282*) and a hemolysin activator-related protein (*ECs1283*). Besides these cytotoxins, one next neighboring strain-specific island (location: 1246040–1310725) is rapidly identified applying inGeno. It contains genes that are known to produce Shiga-toxin2 (stx2: *ECs1205-1206*) [25]. They are involved in pathogenesis and have therapeutic implications as causes for the hemolytic uremic syndrome [26]. Lipid metabolism genes are identified by inGeno as well in this strain-specific island, i.e., a holo acyl-carrier protein (*ECs1284*), an oxoacyl-(acyl-carrier protein) reductase (*ECs1285*), a hydroxydecanoyl-(acyl-carrier protein) dehydratase (*ECs1286*), an acyl-carrier protein (*ECs1287*), an aminomethyl transferase (*ECs1288*) and an oxoacyl-(acyl-carrier protein) synthase (*ECs1289*) are involved. They potentially enable *E. coli *O157:H7 to produce fatty-acid-containing molecules. Furthermore, several neighboring genes are highlighted. These encode urease components (*ECs1321-1327*). They extend the urea cycle and catalyze urea to be transformed into ammonia and carbon dioxide. A number of detoxification proteins (tellurium resistance proteins) are readily visualized in this island by inGeno and the detailed information provided by inGeno is listed in Table 1.

**Table 1:** Genes involved in the strain-specific island of E. coli O157:H7

**Locus**	**Annotation**
ECs1272	Rtn-like protein
ECs1273	FidL-like protein
ECs1274	putative transcriptional regulator
ECs1275	putative oxidoreductase
ECs1276	putative chaperone protein
ECs1277	putative outer membrane protein
ECs1278	putative outer membrane usher protein
ECs1279	putative chaperone protein
ECs1280	putative major pilin protein
ECs1282	hemagglutinin/hemolysin-related prote
ECs1283	hemolysin activator-related protein
ECs1284	putative holo- [acyl-carrier protein] synthase
ECs1285	putative 3-oxoacyl-(acyl carrier protein) reductase
ECs1286	putative (3R)-hydroxymyristol-(acyl carrier prot.) dehydratase
ECs1287	putative acyl-carrier-protein
ECs1288	putative aminomethyltransferase
ECs1289	putative 3-oxoacyl- [acyl-carrier-protein] synthase synthase
ECs1321	urease-associated protein Ure
ECs1322	urease gamma subunit
ECs1323	urease beta subunit
ECs1324	urease alpha subunit
ECs1325	urease accessory protein UreE
ECs1326	urease accessory protein UreF
ECs1327	urease accessory protein UreG
ECs1351	putative tellurium resistance protein TerZ
ECs1352	putative tellurium resistance protein TerA
ECs1353	putative tellurium resistance protein TerB
ECs1354	putative tellurium resistance protein TerC
ECs1355	putative tellurium resistance protein TerD
ECs1356	putative tellurium resistance TerE
ECs1358	putative tellurium resistance protein TerF

**Genes involved in the strain-specific island (selected region in Figure 3)**. This table is an excerpt from an inGeno report. All genes belong to a selected strain-specific island of the *E. coli *O157:H7 genome (location: 1337361–1456555). The annotations marked "unknown", "hypothetic protein" are also included in the inGeno report but not shown for this Table to save space. Similarly, there are a number of "transposases" detected by inGeno, they are excluded from the Table for the same reason.

## Conclusion

InGeno is a user-friendly, platform independent application. It identifies orthologs, visualizes and inspects genome comparison results in good quality. InGeno can be applied for genome comparisons between various strains, closer and less related species. Its graphical output reveals evolutionary changes, bacterial pathogenicity island and differences in metabolism. Furthermore, annotation search capabilities including logical concatenation of keywords, automatic comparison reports and lists offer further information to the user.

## Availability and requirements

• Project name: inGeno

• **For users **we include [see Additional file 3] the linux and windows version of inGeno as well as [see Additional file 2] the required input Files sample_snapshot for the figures visualized in the paper.

• Project homepage: 

• Operating Systems: Windows, Linux, General Unix, Macintosh

• Programming Language: Java

• Other Requirements: Java Runtime Environment 1.5 or higher

• License: GNU GPL

## Authors' contributions

CL: programming and testing of the genome editor/examples, writing of the ms.

TD: advice, organisation and guidance of the study, writing of the ms.

Both authors read and approved the final ms.

## Supplementary Material

Additional File 1 Supplementary information. Tutorials, screen shots and detailed further information on inGeno Click here for file

Additional File 2 sampledata.rar. Sample file to directly start inGeno Click here for file

Additional File 3 ingeno_small.rar. Software. Click here for file
