# Supplementary material for: inGeno – an integrated genome and ortholog viewer for improved genome to genome comparisons
Source: BMC Bioinformatics. 2006 Oct 20;7:461. doi: 10.1186/1471-2105-7-461 (PMC1635569; doi:10.1186/1471-2105-7-461)
Supplement: Additional File 1 — Supplementary information. Tutorials, screen shots and detailed further information on inGeno [file 1471-2105-7-461-S1.doc]

# InGeno Supplementary material

In this supplementary material most figures are obtained from the comparison between *L. monocytogenes* and *L. welshimeri*. They are suitable for illustrating bacterial pathogenicity regions, protein adaptations, e.g., novel-recruited metabolic pathways. However, since the *L. welshimeri* genome has not been published so far, we can not provide its complete genome file as an example. Instead, we use as example the genome file from *L. innocua*. This genome is more similar to *L. monocytogenes*, thus there are no large strain-specific islands. However, using **inGeno**, all genes encoding proteins related to bacterial pathogenicity, e.g., internalins or regulators forming a prfA-box are identified and visualized.

### Step by Step

InGeno provides two methods to import data. The following figures show a routine method (step 1), which allows users to import genbank sequences and their BLAST reports. The other method allows users to reproduce past comparisons using snapshots (step 2). We suggest that visitors begin with step 2 to acquire a quick impression of this software.

- - 1. **Data import (routine pathway)**
       1. **upload sequences**

Users can import sequence files either by the menu “**File-Import Data”** (Figure 1), or by a
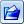
 button.


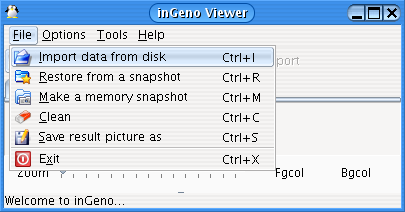


Figure 1: An “Import Data” menu

Afterwards a dialog will appear on screen, where users could specify the file locations (Figure 2).


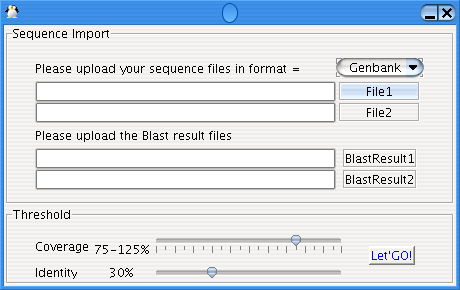


Figure 2: A “sequence import” dialog

The original sequence files for a comparison should be uploaded using “**File1**” and “**File2**” buttons (right side), e.g., the sample sequence files “**lmo.genbank**” and “**lin.genbank**“ can be imported here. Besides genbank, other formats are supported as well, i.e., GenbankXML, Embl and Swissprot (they are listed in the upper combo-box). The lower two buttons (“**BlastResult1**” and “**BlastResult2**”) are designed to upload BLAST reports. Different sequence similarity search programs are supported, such as BLAST and Smith-Waterman search. For instance, our example files (“**lmolin.report**“ and “**linlmo.report**“) were generated by an accelerated version of the Smith-Waterman program (on a Paracel Genematcher). We recommend bidirectional alignments, since InGeno is able to validate the results against each other (in particular to show only the true orthologs).

- - - 1. **threshold**

The lower two sliders allow users to specify the threshold to define orthologs for *in silico* determination by InGeno using the two Blast output files. Two thresholds determine this: The recommended settings (default settings) are 75% alignment coverage and over 30% identity rate to reliable identify true orthologs (proteins predicted to have the same function) and no paralogs (proteins predicted to have only a similar function).

- - 1. **Alternative method to import data (by a snapshot).**


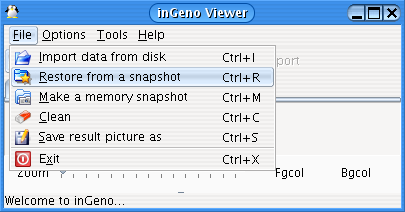


Figure 3: A "Restore from a snapshot" menu

Step 2 is a subroutine for snapshot restoration from a past comparison. Moreover, it provides a method for visitors to test this software rapidly.

Users can download one of the sample snapshot files from the inGeno website, e.g., “**lmolin.snapshot**“, and recover it using the “**restore from a snapshot**” menu item (Figure 3). Moreover, InGeno provides a function to store all the data as a snapshot file. This operation is labeled “**Make a memory snapshot**” in the upper menu (Figure 3).

- - 1. **Dot-plot Analysis**

A dot-plot analysis re-validates the orthologs and strain-specific genes, before a correlation comparison is performed in step 4. The revalidation eliminates false positive homologous pairs and secures that only genes with the highest similarity scores both in the sequence comparison file from genome 1 to genome 2 and in the sequence comparison file back from genome 2 to genome 1 are declared as orthologs (predicted to be proteins with the same function) whereas paralogs are removed (predicted to be proteins with only related function; lower score then another gene in at least one of the two comparisons).


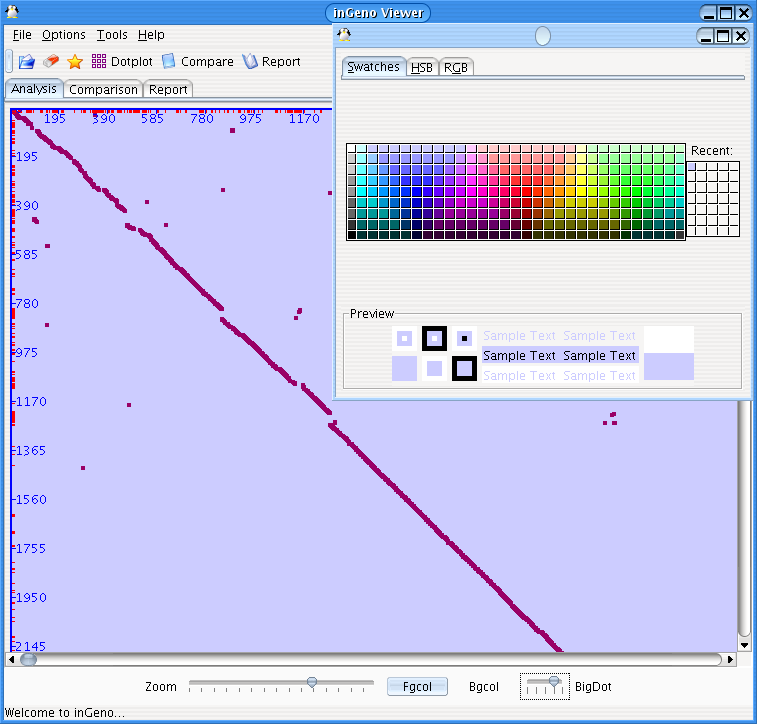


Figure 4: A dotplot-analysis interface

Users click the “**Dotplot**” button in the upper panel to invoke the dot plot analysis. The lower control panel (Figure 4), includes a “**Zoom”** slider to zoom the figure into an appropriate scale. A “**Fgcol**” (foreground color) button and a “**Bgcol**” (background color) button specify the fore- and back-ground colors respectively. A “**BigDot”** slider enlarges the dots in the plot but not the overall scale of the plot.

- - 1. **Comparison interface**

Users can invoke a correlation comparison by clicking the “**Compare**” button. InGeno will perform a linear regression between the identified regions from step 4 and plot the generated line (in red color) on the screen (Figure 5).


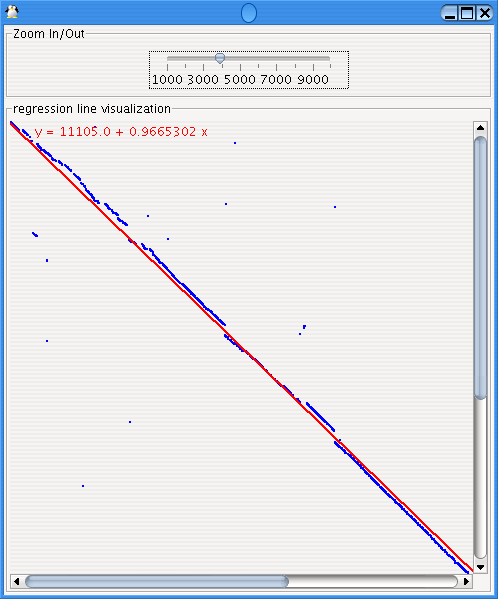


Figure 5: A linear regression line result

- - 1. **Interactive viewing interface**

The next figures illustrate the viewing interface by screenshots from the comparison of the genomes of *L. monocytogenes* with *L. welshimeri*. Interactive control panels help the user to obtain an optimal view. The lower panel contains a couple of sliders, scroll bars and buttons. The scroll bars “**Genome1**” and “**Genome2**” shift the positions of the first or the second genome in the horizontal direction. The “**Distance**” slider shifts the lower genome vertically. A “**Zoom**” slider enlarges or shrinks the comparison view.

To show the legend of the different colors used in the plot, there is a legend box below the comparison map (Figure 6). Functional categories for the different proteins from both genomes appear by clicking the button “function” (see “Functional categories”).

**Linkage lines**

Similar genome regions are visualized as “**locus collinear blocks (LCB)**”. LCBs are determined by the distances between the coordinate of each orthologue and the regression line between the two genomes (from step 4). Consecutive genes with similar distance to the regression line are gathered into LCBs (“gene clusters”). The user can choose 2 colors (to see similar genome regions vs. major rearrangement) or 7 rainbow colors to color the linkage lines. In the latter, linkage lines with similar distance to the regression line get the same rainbow color (regression mode: for closely related genomes; the default mode): Red linkage lines denote close synteny between both genomes, blue ones major rearrangement. The user can determine at which minimal nucleotide distance linkage lines are colored (slider “**threshold**”, lower right corner of the control panel) the threshold value k is the logarithm of the distance to the regression line. Alternatively, linkage lines are rainbow colored (same log distance color scheme, see below) according to the absolute distance shift they have in the two compared genomes to each other (absolute mode: best for more distantly related genomes). The two different modes can be switched in the “option / mode” menu. The slider “**hide**” allows users to have distant linkage lines to be hidden (this uses again the same logarithmic distance scale).

The coloring function is summarized as follows:

Regression mode:

**K** = log ( ***distance*** ) – ***threshold***

*threshold* is a number between 0 and 7.0 (corresponding to distances between 1 and 10 million base pairs), which is given by the slider in the lower right corner of the panel.

*distance* is the decadic logarithm of the distance to the regression line from the plot.

*e.g,* the regression line is determined as *y* *=* *a + bx,* the coordinate of the plot is (x,y), so that, the distance is calculated by

______

***distance*** = (b * x – y + a) / √ b^2^ *+* 1

Absolute mode:

**K** = log ( ***absolute distance shift between genes*** ) – ***threshold***

*threshold* is a number between 0 and 7.0 (between 1 and 10 million base pairs), which is given by the slider in the lower right corner of the panel.

The linkage line color is determined by the “**K**” value (Table 1),

| **K** | < 1 | [1,2[ | [2,3[ | [3,4[ | [4,5[ | [5,7[ | ≥7 |
| --- | --- | --- | --- | --- | --- | --- | --- |
| **Color** | Red | magenta | yellow | green | blue | grey | black |

Table 1: Coloring scheme for the linkage lines.

In addition to the standard rainbow colors, inGeno provides “**Reverse**” colors as an option.

As the two genomes compared in Fig. 6 are relatively close in evolution, there are no blue lines present in the figure. However, green lines state the corresponding genes have undergone some rearrangement. Yellow color declares that the region is relatively conserved in comparison to others but there is no very close synteny (Figure 6-7, 11-12).


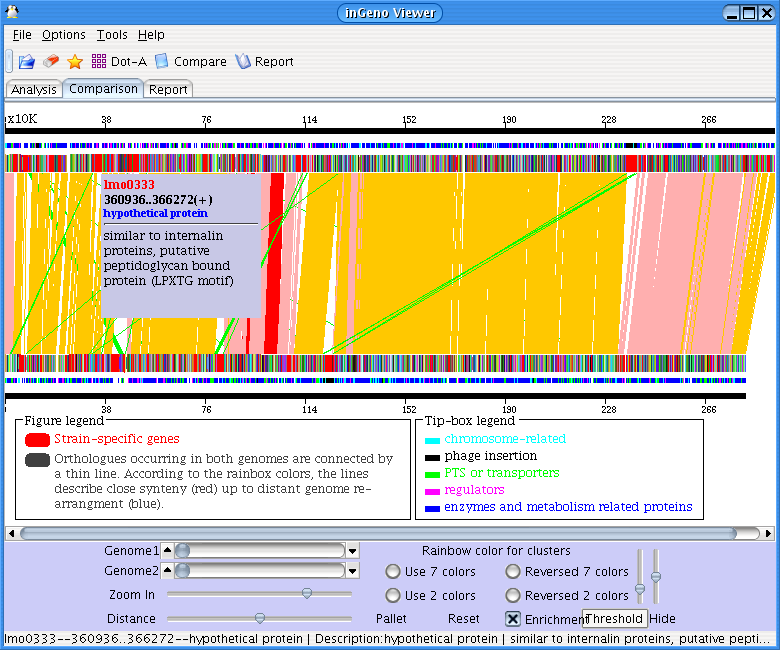


Figure 6: A user-friendly interactive interface for genome comparisons

The genomes of *Listeria monocytogenes* (lower genome in the figure) and *Listeria welshimeri* (upper genome) are compared using InGeno. Each pair of orthologs is shown as an ellipsoid and colored with the same randomly chosen color in both genomes (the threshold for *in silico* determination of orthologs is specified during data importation; see above, Figure 2). All strain specific genes are colored in red.


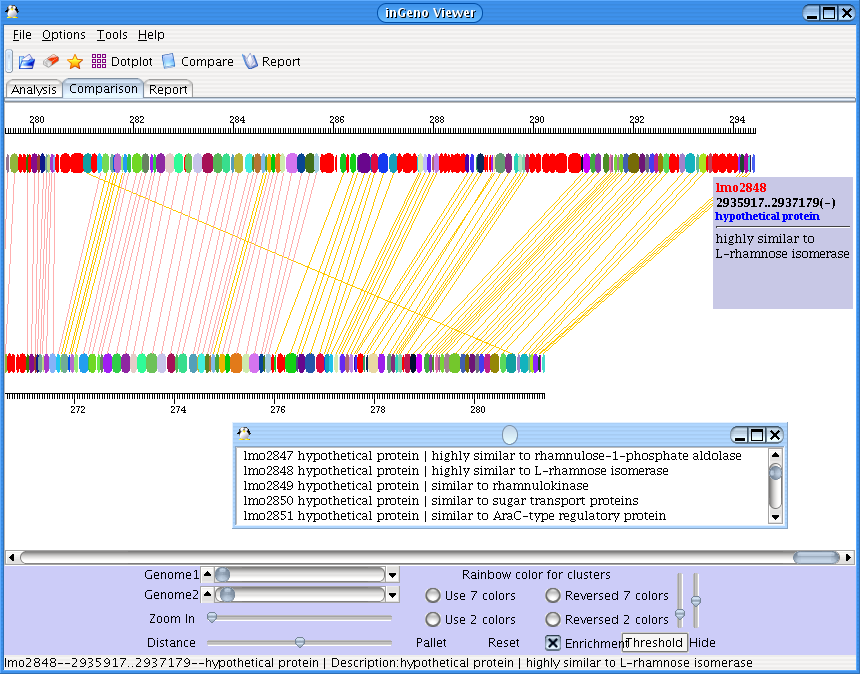


Figure 7: A screenshot showing strain-specific gene islands

Several strain-specific gene islands are indicated in Figure 7. As an example, the gene cluster beginning with ***lmo2848*** can be observed and investigated (close to the right / 3’ end of the screenshot). Its associated annotation report declares it as a **rhamnose isomerase**, the next gene ***lmo2849*** is a putative **rhamnulokinase** (Figure 8), ***lmo2850*** encodes a **sugar transport protein**. This may lead to different carbon-source utilization in *L. monocytogenes* compared to *L. innocua*. The genes probably form an operon.


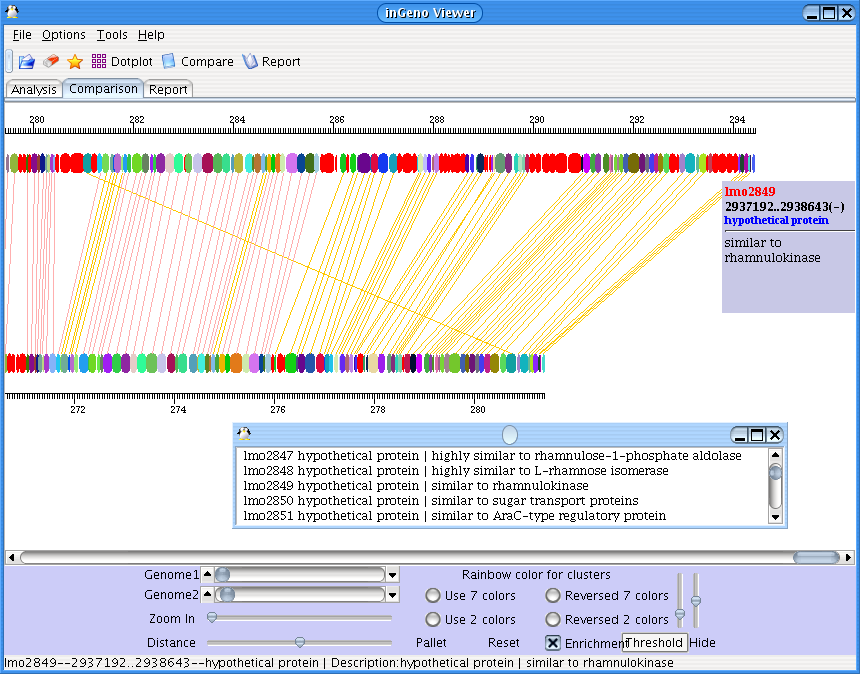


Figure 8: A screenshot of strain-specific island investigation (gene lmo2849)

In order to investigate a gene island, users can perform a “**batch selection**” using a mouse operation of “drag-and-release”. A rectangle will appear, denoting the selection region (Figure 9). Figure 10 shows the generated report using this batch selection. The upper part is the list of genes and their annotation involved in this island, the lower table lists keywords and top key word frequencies automatically generated from the genome annotation by inGeno.


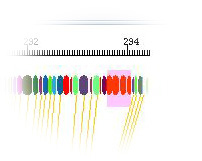


Figure 9: A batch-selection of continuous genes


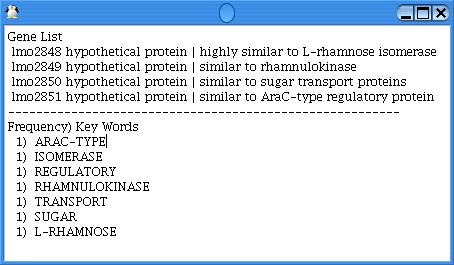


Figure 10: A list of selected genes and their keywords

**Genome rearrangement events** These can be visualized and analyzed using InGeno as well (Figure 11). Green linkage lines indicate a region where a transposition occurs. In the example this may be caused by transposons, due to a number of transposases present in *L. monocytogenes*. Variant color of linkage lines denotes different distance to the genome regression line (see above). Figure 12 illustrates the comparison between two closely related genomes (*E. coli* K-12 W3110 vs MG1655). It is apparent that most genes and their positions are highly conserved, except for a large inversion island.


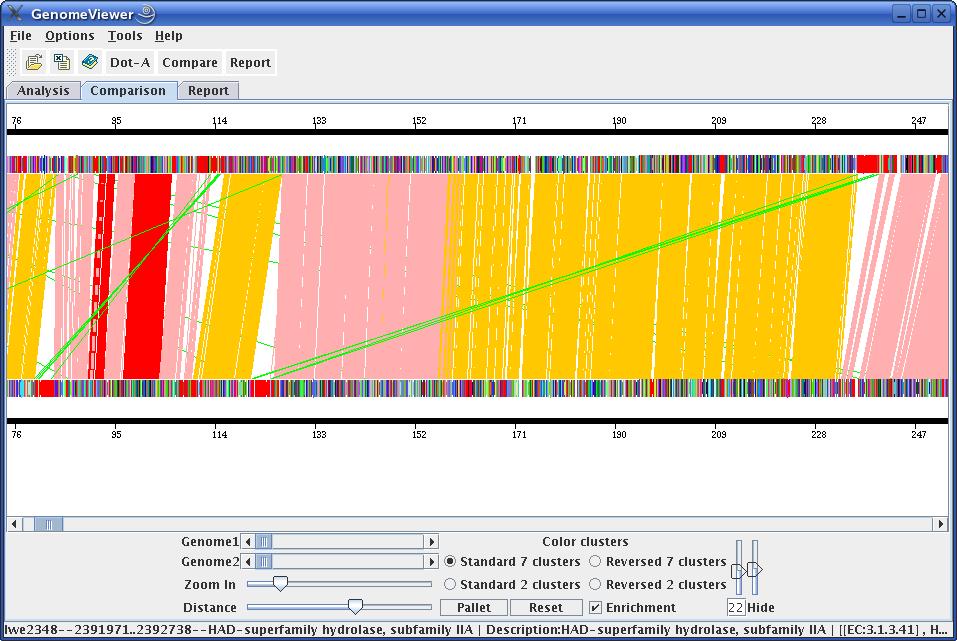


Figure 11: An example demonstrating locus collinear blocks and genome rearrangement events


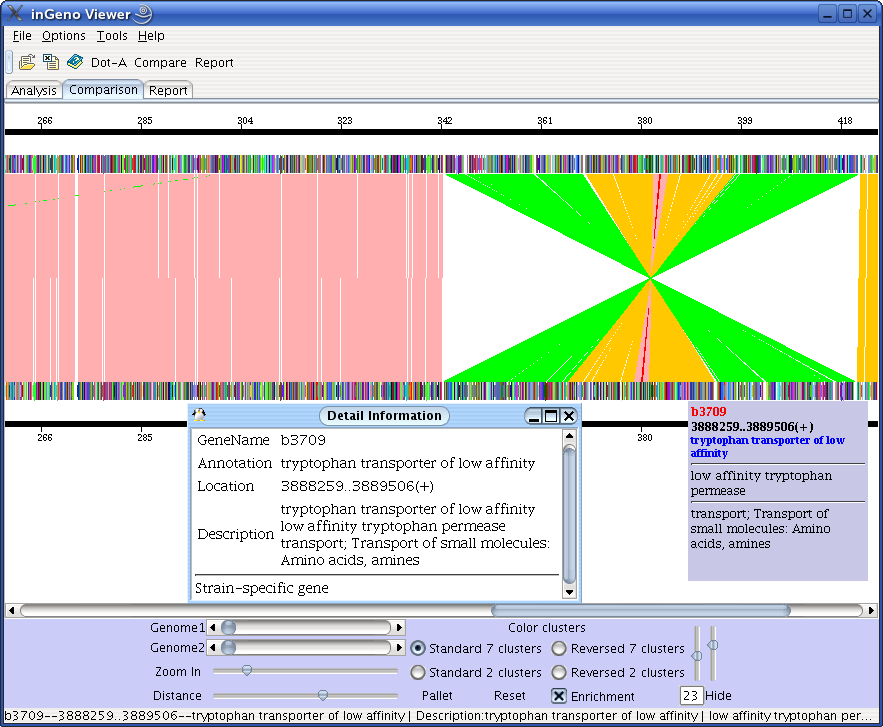


Figure 12: A screenshot of genome comparison between closely related strains

**Functional categories.** InGeno assigns functional categories according to genome annotation. Keywords from the annotation are filtered out by the “**function**” subroutine and painted as tiny color rectangles. Once users invoke the option “function” (Figure 13; in the top menu bar), tiny rectangles will appear in color indicating functional categories of the corresponding genes, e.g., blue (enzymes or metabolism-related proteins), cyan (chromosome-related), pink (regulators), green (Phosphor-Transfer System or other transporters) and black (phage insertion). A legend explains all different colors for these functional categories.


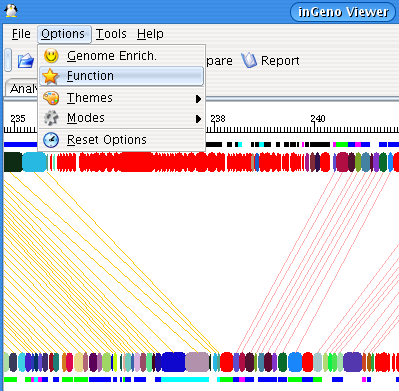


Figure 13: Gene function (visualized by tiny color rectangles close to the genome)


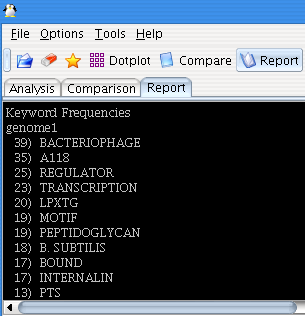


Figure 14: A strain-specific gene report

- - 1. **Report**

When the “**Report**” button is clicked (Figure 14), the program will summarize all the strain-specific genes, list their annotations and sort their keywords by their frequencies. Users can thus acquire the information of strain characteristics, e.g., in the *L. monocytogenes* report, the top lines (top frequency keywords) are Bacteriophage A118, LPXTG motif, Peptidoglycan, *B.Subtilis*, Internalin. They coincide with the fact, that *B.subtilis* is one of its closest species and the surface-associated (seldom secreted) LPXTG-motif proteins, Internalins are deeply connected to bacterial pathogenicity.

- - 1. **Annotation browser**

Quite helpful in our experience are the InGeno routines which allow also logical operations (AND, AND NOT, OR) between annotation keyword to create more specific reports from a large genome annotation file.

InGeno provides for this an annotation browser. A regular expression can be inserted in the upper text-field (Figure 15), e.g., “internalin AND not putative AND not hypothetical OR kinase AND not putative AND not hypothetical”. Such a regular expression includes one or more interesting keywords, which are concatenated by "AND", "OR" and "NOT" to describe their logical relation.

The text-field is case insensitive. The keywords and the logical oparators must have at least one white space (" ") between them. All redundant spaces will be ignored by the software. InGeno allows users to add logical operators by buttons (the buttons "**AND**", "**OR**", "**NOT**" closely below the text-field). The button "**Reset**" can clear the input and the button "**Back**" provides a history function. The latter allows users to trace back the previous keyword and regular expression searches they performed before. The combo-box named "show lines" allows users to change the number of lines listed in the result box, e.g., 20 lines are selected in the figure.


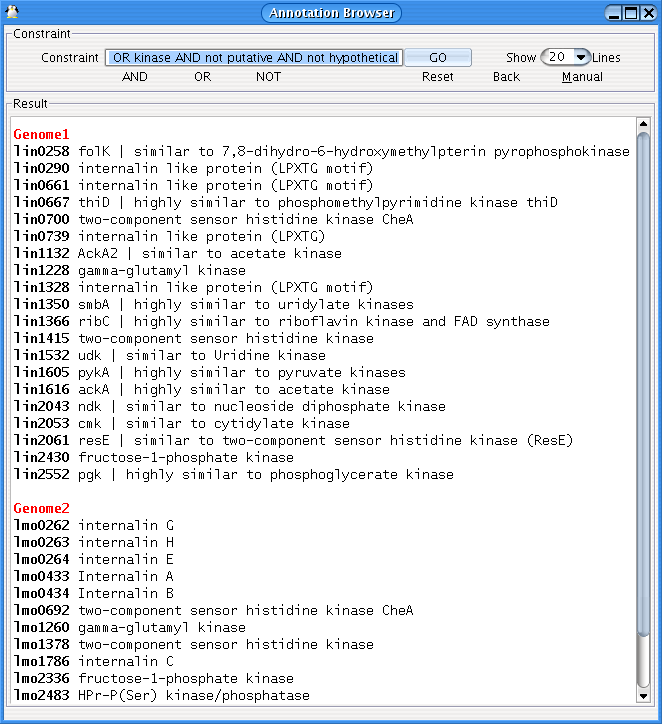


Figure 15: A screenshot of the annotation browser.

The logical priority between the operators is listed in the table below. "NOT" gets always the highest priority (it describe a relation that the given keyword must be absent in the annotation9. “AND” has middle priority.

| *Logical operator* | OR | AND | NOT |
| --- | --- | --- | --- |
| *Priority* | Low | Middle | High |

Table 2: Priorities for different logical operators.

- - 1. **A “GenomeToProteome” Tool**

The routine “GenomeToProteome” (Figure 16) provides a convenient way to obtain proteome files from original genome sequences. Users can upload the genome sequence (button “**File**” in the upper panel) to upload the genome file (please specify an appropriate format in the right combo-box). InGeno automatically parses the file to read the genome position. However, users should specify standard keywords used in the genome file to indicate gene positions, for example, the keyword (tag) “gene” in the “**Specified Tag**” box to indicate that all gene position are specified by the tag “/gene=”lmo2637” (this then gives accurate positions of the ORFs for the parser, so that not the whole genome is translated; the Genbank tags are easily recognized by the conversion program). Next click the “**Convert**” button and within seconds the translation of all the ORFs is collected in a multi-FASTA format file. Such genome files are for instance suitable for BLASTP or Smith-Waterman (S-W) algorithm runs and other types of differential genome analysis or large-scale comparisons of proteome data requiring the predicted proteome from the genome sequence.

*
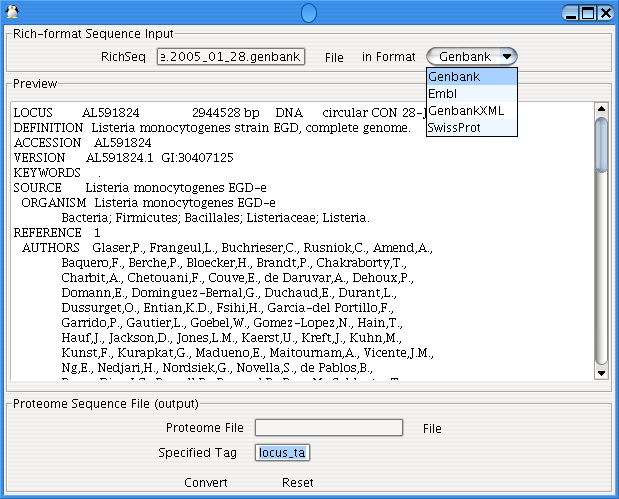
*

Figure 16: An integrated "GenomeToProteome" sequence conversion tool

### Q&A

- - 1. **How to obtain and prepare the input data required by inGeno?**

Normally the software requires four input files to generate an ideal comparison map, they are the files of

A) Genome1 sequence

B) Genome2 sequence

C) Genome1 vs Genome2 BLAST report

D) Genome2 vs Genome1 BLAST report

A and B are either from a web database of GenBank, EMBL, DDJB, or a private annotation source, whose sequence formats should be readable by inGeno.

C and D are pairwise comparison reports, generated by BLASTP kind of comparisons (NCBI-BlastP or the Smith-Waterman algorithm is recommended). Users can apply our sequence conversion tool (see Figure 16 “genometoproteome”) to obtain all translated protein sequences when importing a genome file (A or B), inGeno will rapidly parse it and collect its encoded protein sequences using a multi-FASTA-format. The generated file can be used as an input for BLAST programs. NCBI-BLASTP is relatively faster than S-W algorithm, whereas S-W is more strict and precise. Another appropriate choice is the Paracel GeneMatcher accelerated version of S-W program, it is capable to complete a same task in minutes and achieve a similar level of accuracy. Originally inGeno was written specially for the programs using the S-W algorithm, to obtain a top quality comparison.


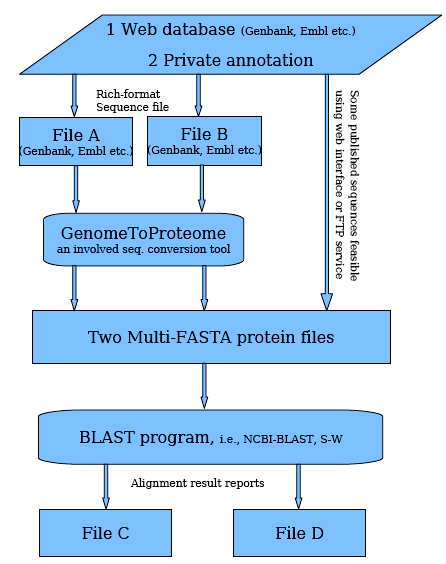


Figure 17: A working flowchart of data preparations.

As a summary, different procedures for data preparation are illustrated in Figure 17.

- - 1. **How to apply the “BLAST” software to analyze the genome data and obtain the comparison reports?**

A popular alignment software package, NCBI-BLAST, is highly recommended for most users, in particular there is only an ordinary PC available nearby. A tutorial is located at:

<http://www.ncbi.nlm.nih.gov/blast/docs/blast.html>

The procedures involved are

- Downloading the software package from

[ftp://ftp.ncbi.nlm.nih.gov/blast/executables/release](ftp://ftp.ncbi.nlm.nih.gov/blast/executables/release/)

- Unpack it and set the required environment.
- Format a sequence database.

*e.g.,* ***formatdb -i*** *lmo* ***-p*** *T*

*here, lmo is a multi-FASTA file containing protein sequences,*

*the parameter “-p” specifies that the input is protein sequence.*

- Perform a BLASTP run

*Type:*

***blastall******-p*** *blastp* ***-i*** *lin* ***-d*** *lmo* ***-o*** *linlmo.report* ***-e*** *0.000001* ***-M*** *BLOSUM62*

*explanation of the qualifiers:*

*-I lin is the query data, -d lmo is the queried database, -o specifies output report filename, -e states E-value threshold, -M chooses a matrix which will be used during the calculation.*

*-p specifies the name of the BLAST program, here it is blastp.*

Paracel Genematcher2 provides an accelerated version of the Smith and Waterman algorithm. It is powerful to correctly identify orthologous genes. The main procedure is

- Prepare a fdf file system and format a database by the commands

***btk db load src=****<datafile>* ***dst=****<location>* ***seqtype=****protein*

- Compare them against each other using the SWP program.

***swp*** ***q=****<query>* ***d=****<database location>* ***output=****<report file>* ***sort=****evalue* ***evalue_threshold=****<E-value>* ***format =blast0*** ***matrix=****BLOSUM62*

The format qualifier ”blast0” is recommended, as it is one of the most stable formats for the Genematcher system (in particular regarding well formatted output for further analysis, see below)

- - 1. **Why does inGeno not parse my S-W alignment report from GeneMatcher?**

Paracel GeneMatcher alignment reports may have incomplete information, e.g. unexpected loss of gene names, wrong new-line characters. By comparison of different report formats, we concluded that **BLAST0** format is the best choice, which contains the fewest mistakes. However, even in this format, the generated report misses protein names in rare frequency. Thus users have to fix them manually.

- - 1. **Why does inGeno not read my sequence files in EMBL format?**

Firstly please make sure the sequence type is specified during importing the sequence files, because the default choice there is Genbank format. Secondly, the EMBL format sequence file might need to be modified, since we found some EMBL sequence files are not very standard, containing some contexts can not be read by BioJava, e.g., unbalanced quotes, missing tags and wrong chars which result in BioJava exceptions. We are expecting that the coming version of BioJava can handle these perfectly.

- - 1. **Contact for more information**

Phone us or mail us please, you can find detailed information in the web-page, we are looking forward to hearing from you.

Chunguang Liang (techn.), Phone: 0049-931-888-4561 (Fax: -4552)

E.mail: [liang@biozentrum.uni-wuerzburg.de](mailto:liang@biozentrum.uni-wuerzburg.de)

Prof. Dr. Dandekar, Phone: 0049-931-888-4551 (Fax: -4552)

E.mail: [dandekar@biozentrum.uni-wuerzburg.de](mailto:dandekar@biozentrum.uni-wuerzburg.de)

- - 1. **How to report bugs?**

Please mail us via [liang@biozentrum.uni-wuerzburg.de](mailto:liang@biozentrum.uni-wuerzburg.de), we appreciate that you could help us to improve the software.
